# Intravenous immunoglobulin is safe and effective in controlling pre-existing paraneoplastic neuromuscular diseases in cancer patients treated with immune checkpoint inhibitors: two case reports and literature review

**DOI:** 10.3389/fonc.2023.1199195

**Published:** 2023-07-03

**Authors:** Ge Xiong, Richard Benjamin Young, Helen Chow, Emanual Maverakis, Ricardo A. Maselli, David Paul Richman, Tianhong Li

**Affiliations:** ^1^ Department of Neurology, School of Medicine, University of California, Davis, Sacramento, CA, United States; ^2^ Division of Hematology/Oncology, Department of Internal Medicine, School of Medicine, University of California, Davis, Sacramento, CA, United States; ^3^ Department of Dermatology, School of Medicine, University of California, Davis, Sacramento, CA, United States

**Keywords:** immune checkpoint inhibitor, intravenous immune globulin (IVIg), safety and effectiveness, pre-existing, paraneoplastic neurologic disease

## Abstract

Immune checkpoint inhibitors cause rare but potentially fatal neuromuscular complications, leading to a concern to use these agents in cancer patients with pre-existing autoimmune or inflammatory neuromuscular diseases. We report two such patients with paraneoplastic dermatomyositis and “seronegative” paraneoplastic demyelinating neuropathy, respectively, who have been successfully treated with immune checkpoint inhibitor monotherapy as well as maintenance intravenous immunoglobulin. While controlling the paraneoplastic or autoimmune neuromuscular diseases, the use of intravenous immunoglobulin did not compromise the anti-cancer effect of immune checkpoint inhibitor.

## Introduction

Immune checkpoint inhibitors (ICIs) have revolutionized cancer therapy by activating immune responses through releasing the blockage to programmed cell death protein-1 (PD-1) or cytotoxic T lymphocyte associated antigen 4 (CTLA-4) signaling pathways. The unleashed immune responses can cause a variety of autoimmune syndromes in cancer patients receiving ICIs. ICI-associated neuromuscular complications, which include polymyositis, dermatomyositis, myasthenia gravis, Guillain–Barre syndrome (GBS), and chronic inflammatory demyelinating neuropathy (CIDP), are rare but potentially fatal. These neuromuscular complications not only cause high mortality but also lead to significant morbidity including paralysis and imbalance (2.9%–4.2%) (1, 2). There is an unmet need for neurologists and oncologists to develop a clinical workflow for early recognition and effective treatment of these ICI-associated neuromuscular complications. Patients with preexisting autoimmune diseases have increased risks for developing cancers (3–5). It is unknown if patients with preexisting inflammatory myopathy/neuropathy can safely receive or continue immunomodulatory agents and if the immunomodulatory agents may compromise the efficacy of ICI against cancer. Here, we report two cases of preexisting autoimmune paraneoplastic myopathy/neuropathy that were successfully controlled with intravenous immunoglobulin (IVIG) while their cancers remained in clinical remission due to PD-1 inhibitors (**Table 1**).

**Table 1:** Summary of two cases of inflammatory neuromuscular diseases.

	Patient 1	Patient 2
**Age at onset (years), sex**	66, male	55, female
**Initial lab results**	Elevated CK (885 U/L), ESR (59 mm/h), positive TIF-1 gamma, anti-p155/140 antibodies Normal B12, TSH, SPEP, CRP, ANA, SSA, SSB, paraneoplastic antibody panel, A1C	Elevated ESR (85 mm/h) Normal B12, TSH, SPEP, ganglioside antibodies, MAG antibody, ANA, dsDNA antibody, rheumatoid factor, paraneoplastic antibody panel
**Initial CSF results**	Not performed	Elevated protein (275 mg/dl), normal white blood cell count (1/mm^3^)
**Initial electrophysiological study**	Not performed	Chronic inflammatory demyelinating polyneuropathy (CIDP, meeting EFNS/PNS criteria)
**IVIG treatment course** **Response to IVIG treatment only**	0.4 g/kg/day × 5 days every 4–6 weeks for 11 months Not available	2 g/kg once then 1 g/kg every 4 weeks for 22 months, then 1 g/kg every 8 weeks for 21 months Worsening motor and sensory deficits
**Interval between neurological symptoms and cancer diagnosis (months)**	2 months	18 months
**Cancer pathology**	Metastatic poorly differentiated lung adenocarcinoma	Extraperitoneal malignant melanoma (M1a/metastatic melanoma)
**Routine cancer treatment/duration**	None	Dabrafenib 150 mg twice daily + Trametinib 2 mg daily for 10 months
**ICI treatment**	Pembrolizumab every 3 weeks for 21 cycles	Nivolumab every 4 weeks for 24 cycles

ANA, anti-nuclear antibody; CK, creatine kinase; CRP, C-reactive protein; CSF, cerebrospinal fluid; dsDNA, double-strand deoxyribonucleic acid; ESR, erythrocyte sedimentation rate; MAG, myelin-associated glycoprotein; SPEP, serum protein electrophoresis; SSA, Sjogren’s syndrome-related antigen A autoantibody; SSB antibody, Sjogren’s syndrome- related antigen B autoantibody; TIF-1, transcription intermediary factor-1; TSH, thyroid- stimulating hormone.

## Case description

**Figure 1:**
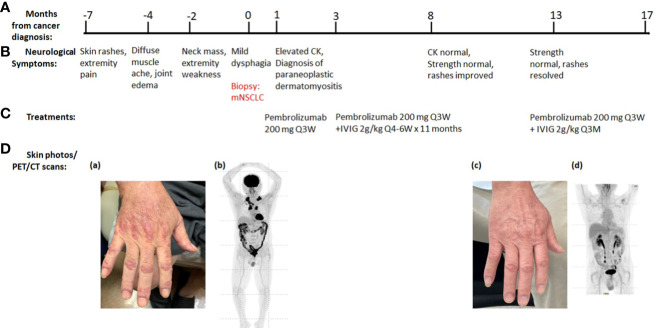
Summary of key events in the clinical course of patient 1. **(A)** Timeline from the cancer diagnosis. “0” is the time when metastatic non-small cell lung cancer (mNSCLC) was confirmed by the biopsy. **(B)** Timeline of neurological symptoms. **(C)** Summary of the systemic treatments. **(D)** Serial skin photos and PET images assess disease status. (a) Extensive Gottron’s papules in hands before ICI treatment. (b) PET before ICI treatment showed increased radiotracer uptakes in lung, cervical, and mediastinal lymph nodes. (c) Resolution of skin rash after ICI and IVIG treatment. (d) Resolution of all FDG-avid tumor lesions after ICI treatment.

**Patient 1:** A 66-year-old Asian man, former smoker (29 pack-year history of cigarette smoking, quit 15 years ago) with controlled hypertension and diabetes, presented with rash and muscle ache. The erythematous papular rash was first present in the extremities, head, and trunk, accompanied by pruritus and burning pain, which worsened after sun exposure (**Figure 1D** (a)). Over a 3-month period, the symptoms rapidly evolved into diffuse muscle pain, edema, and pain in wrists, elbows, and knees, along with weakness in the lower extremities, especially noticeable in climbing stairs. He was initially treated with hydrocortisone 2.5% lotion and ketoconazole 2% shampoo without improvement in his symptoms. Two months later, the patient noticed multiple lateral cervical masses, which enlarged over the subsequent 3 months, in association with sharp pain radiating from the neck to bilateral upper extremities. Over the next month, he noticed weakness progressing to bilateral arms, then difficulty swallowing dry food. Ultrasound of the neck revealed cervical lymphadenopathy. Positron emission tomography/computerized tomography (PET/CT) scan showed a subpleural right upper lobe bilobed primary tumor, scattered left upper lobe pulmonary nodules, too small to characterize by PET, and bilateral cervical and mediastinal lymphadenopathy consistent with metastatic lymph nodes. Brain magnetic resonance imaging (MRI) did not reveal any metastatic disease. Ultrasound-guided core needle biopsy of the left supraclavicular lymph node revealed metastatic poorly differentiated adenocarcinoma of lung primary with 100% expression of programmed death-ligand 1 (PD-L1) by immunohistochemistry stain. There was insufficient tumor specimen for tumor genomic profiling. Next-generation sequencing of plasma circulating tumor DNA did not identify any actionable mutation. The patient was started on pembrolizumab standard-of-care dose at 200 mg intravenously (IV) every 3 weeks and referred to neurology for the neck pain and extremity weakness, and dermatology for the rash. Neurological exam demonstrated weakness in bilateral proximal upper and lower extremities [Medical Research Council (MRC) grade 4/5 in deltoid, biceps, triceps, wrist extension/flexion, hip flexion, and knee extension/flexion], normal deep tendon reflexes, hyperesthesia to pinprick in bilateral upper extremities and thighs, normal vibration, and temperature sensation. Dermatological evaluation showed extensive erythematous, lichenified plaques and papules on extremities, trunk, and scalp. He had positive Gottron’s papules, Shawl’s sign, and V neck erythema (**Figure 1D**). Further testing revealed elevated creatine kinase (CK, 885 U/L), high positive anti-transcription intermediary factor 1 gamma (TIF 1-γ) antibody (154 units), and myositis-specific anti-p155/140 antibody (**Table 1**). Magnetic resonance imaging (MRI) of cervical spine showed mild to moderate degenerative changes at multiple levels without abnormal cord signal or root impingement. The patient refused electromyography/nerve conduction study (EMG/NCS) and muscle biopsy. The neurological diagnosis was paraneoplastic dermatomyositis. He was started on clobetasol 0.05% ointment twice daily that helped his pruritus and burning pain. Two months later, he started IVIG 2 g/kg every 4–6 weeks for the autoimmune disease while taking PD-1 inhibitor pembrolizumab 200 mg IV every 3 weeks (**Figure 1**). After 11 months of combined treatment, the rash resolved (**Figure 1D**), muscle strength returned to normal (MRC grade 5/5), and neck pain resolved. CK level became normal. TIF-γ antibody became low positive (26 units). PET/CT scan demonstrated resolution of essentially all previously active tumor lesions while his neurological symptoms were controlled, so the IVIG was reduced to maintenance dose at 2 g/kg every 3 months while pembrolizumab was maintained at 200 mg IV every 3 weeks (**Figure 1**). At the 24-month evaluation, he remained in clinical remission with metastatic lung cancer with normal muscle strength and no rash. Based on common terminology criteria for adverse events (CTCAE) grading, his function level was grade 3 initially. It improved to grade 0 after combined treatment of IVIG and pembrolizumab.

**Figure 2:**
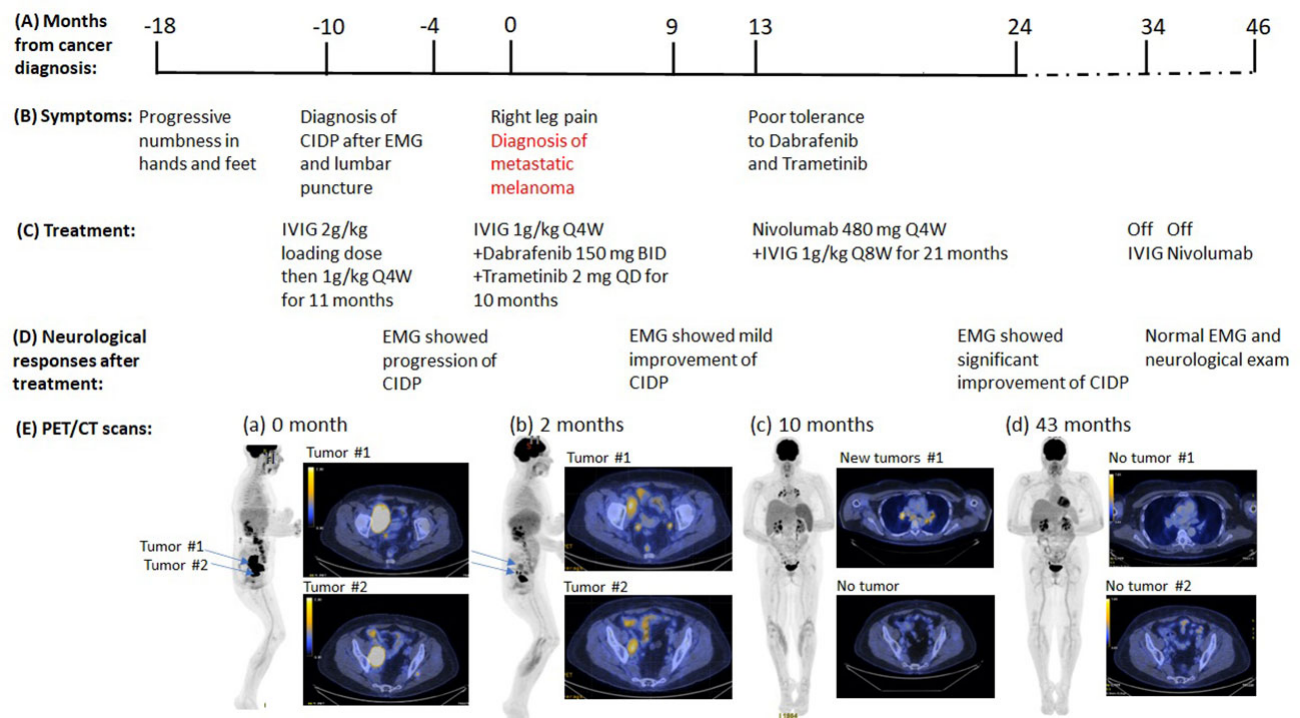
Summary of key events in the clinical course of patient 2. **(A)** Timeline from the cancer diagnosis. “0” is the time when metastatic melanoma was confirmed by the biopsy. **(B)** Timeline of neurological symptoms. **(C)** Summary of the systemic treatments. **(D)** Serial neurological responses after treatments. **(E)** Serial PET images assess disease status. (a) Right pelvic mass (8.5 x 5.4 cm), hypermetabolic right retroperitoneal and iliac/inguinal lymph nodes. (b) to (c), Continued improvement in pelvic lymphadenopathy, but new mediastinal and bilateral hilar lymphadenopathy (d) No evidence of residual, recurrent, or metastatic tumor.

**Patient 2:** A 55-year-old Caucasian woman with past medical history of resected cutaneous melanoma of right leg 27 years ago developed symmetrical ascending numbness that progressed from feet and hands to the thighs and elbows over 3 months. She also complained of intermittent joint pain and fatigue. Neurological examination showed normal strength (MRC grading 5/5), decreased reflexes throughout (1+), decreased vibration sensation at bilateral ankles, and decreased pinprick sensation below bilateral mid-shins. The initial EMG study showed features consistent with an acquired demyelinating sensorimotor polyneuropathy with active denervation in right tibialis anterior muscle. Cerebrospinal fluid (CSF) studies demonstrated albuminocytological dissociation [elevated protein 275 mg/dl and normal white blood cell (WBC) count 1/mm^3^]. Additional tests were normal, including vitamin B12, thyroid-stimulating hormone (TSH), serum protein electrophoresis, ganglioside antibodies, anti-myelin-associated glycoprotein (MAG) antibody, paraneoplastic antibody panel [including anti-Hu, collapsing response mediator protein-5 (CV2/CRMP-5), ganglionic acetylcholine receptor, amphiphysin antibody, glial nuclear antibody, voltage-gated potassium channel antibody, P/Q-type calcium channel antibody, and Purkinje cell cytoplasmic antibody], antinuclear antibodies (ANA), anti-double-stranded DNA antibody, and rheumatoid factor, except for elevated erythrocyte sedimentation rate (ESR) (85 mm/h). Her clinical presentation, EMG/NCS study, and CSF findings led to a diagnosis of CIDP based on European Federation of Neurological Societies/Peripheral Nerve Society EFNS/PNS criteria (**Table 1**). She received IVIG infusions, 1 g/kg every 4 weeks for 6 months after an initial dose of 0.4 g/kg/day for 5 days. Although the patient reported improvement of fatigue at follow-up, her neuropathy progressed based on the neurological exam. Examination at 14 months after symptom onset demonstrated mild weakness in toe extension and flexion (MRC grading 4/5), complete areflexia, decreased pinprick in bilateral fingers, and below bilateral mid-shins. Repeat EMG/NCS study (**Table 2**) also demonstrated interval progression of demyelinating sensorimotor polyneuropathy with partial conduction block, decreased amplitudes, and active and chronic denervation in distal extremities. Because the patient did not respond to the standard treatment of CIDP, alternative underlying etiologies were explored, including paraneoplastic neuropathy given her prior history of melanoma. At 18 months after initial onset of these symptoms, she developed right hip and back pain. Pelvic CT scan revealed a large pelvic wall mass and intra-abdominal lymphadenopathy. PET/CT scan showed a hypermetabolic right retroperitoneal soft tissue mass of 8.4 × 3.5 × 8.7 cm and several hypermetabolic right retroperitoneal and iliac/inguinal lymph nodes suggestive of nodal metastasis (**Figure 2**). There were also multiple focal regions of increased radiotracer uptake within the axial and appendicular muscles, which are nonspecific but might represent melanoma metastasis or, atypically, inflammatory changes or sites of subcutaneous lesions. Biopsy of the right retroperitoneal mass confirmed recurrent melanoma, and tumor genomic profiling revealed BRAF V600E mutation. The patient was treated with targeted therapy dabrafenib 150 mg twice daily and trametinib 2 mg daily (**Table 1**, **Figure 2**). She had partial tumor response by PET scan after three cycles of treatment, and the paresthesias were improved as well. Her strength remained stable with mild weakness in toe extension and flexion (MRC grading 4/5). EMG/NCS study after 10 months of targeted therapeutics together with monthly IVIG treatment also demonstrated mild improvement of neuropathy (**Figure 2**, **Table 2**). At that time, dabrafenib/trametinib treatment was discontinued after 10 cycles due to mixed tumor response and poor tolerance. The oncologic treatment was switched to PD-1 inhibitor nivolumab 480 mg IV every 4 weeks. Simultaneously, the frequency of maintenance IVIG was reduced from every 4 weeks to every 8 weeks for 21 months due to the concern that frequent IVIG infusions might reduce the anti-tumor effect of the PD-1 inhibitor nivolumab (**Figure 2**). Repeat EMG testing, after 12 months of dual treatment with nivolumab and IVIG, demonstrated significant improvement. After 21 months of double therapy, neurological examination improved to normal strength (MRC grading 5/5) in all extremities, with normal sensation, and IVIG was discontinued. Surveillance PET/CT scans showed that the patient achieved complete remission (CR) after 19 cycles of nivolumab and has remained in CR since then (**Figure 2**). Nivolumab treatment was discontinued after 24 cycles. At the time of this submission, the patient has been in remission from both melanoma and neuropathy for 12 months (**Figure 2**). Based on CTCAE grading, she was at grade 2 before nivolumab treatment and it improved to grade 0 after the combined treatment of IVIG and nivolumab.

**Table 2:** Serial nerve conduction/EMG tests of patient 2.

Category	Item	Location	Baseline	6 months after IVIG monotherapy	10 months after IVIG+ chemotherapy	12 months after IVIG +ICI	
Sensory nerve action potentials (SNAPs)	Amplitude (μV)	Median	No response	No response	4.2	7.5
		Ulnar	No response	No response	No response	4.2
		Sural	No response	No response	No response	6.2
	Conduction velocity (m/s)	Median	No response	No response	31	40
		Ulnar	No response	No response	No response	36
		Sural	No response	No response	No response	37
Compound muscle action potentials (CMAPs)	Distal latency (ms)	Median	6.35	7.45	4.69	4.32
		Ulnar	6.56	6.72	4.69	3.75
		Peroneal	11.67	13.59	7.71	4.84
		Tibial	10.89	9.53	9.22	4.27
	Amplitude (mV)	Median	5.2	5.3	6.6	6.6
		Ulnar	7.6	6.1 (conduction block at forearm)	12	14.1
		Peroneal	2.1	1.3	1.9	4.1
		Tibial	2.0	0.6	0.9	5.0
	Conduction velocity (m/s)	Median	19	18	28	32
		Ulnar	25	19	28	34
		Peroneal	23	19	25	30
		Tibial	33	22	26	32
F wave Latencies		Median	64.7	ND	26.0	40.9
		Ulnar	66.4	ND	50.7	40.4
		Tibial	89.6	ND	ND	62.2
Needle EMG			First dorsal interosseus	Normal	1+ positive sharp wave Decreased recruitment, prolonged duration	Decreased recruitment, prolonged duration	Decreased recruitment
			Tibialis anterior	1+ fibrillation Decreased recruitment	Decreased recruitment, prolonged duration	Decreased recruitment, prolonged duration	Decreased recruitment, prolonged duration
			Gastrocnemius	Normal	1+ positive sharp wave Decreased recruitment, prolonged duration	1+ fibrillation Decreased recruitment, prolonged duration	Decreased recruitment

All the nerve conduction studies and needle sampling were performed in right upper and lower extremities. Very abnormal nerve conduction studies were labeled in red color; mildly abnormal nerve conduction studies were labeled in blue color; normal nerve conduction studies were labeled in black color. Active denervation changes (fibrillations or positive sharp waves) in needle EMG were labeled in red color to suggest active neuropathy.

EMG, electromyography; SNAPs, sensory nerve action potentials; CMAPs, compound muscle action potentials; ND, not done; μV, microvolt; mV, millivolt; m/s, meters/second; ms, milliseconds.

## Discussion

The risk of progression of an inflammatory neuromuscular disease with ICI treatment is unknown. ICI targets PD-1 or CTLA-4 signaling pathways to disrupt immune regulation, thereby enhancing the anti-tumor immune response by both T cell- and B cell-mediated processes (6, 7). It is possible that the ICI may also activate a subgroup of regulatory T cells that could play a role in the immune-mediated paraneoplastic processes. For the autoimmune disorder myasthenia gravis (MG), the 2020 international consensus guidance considered the potential for exacerbation with ICI treatment—given the propensity for this treatment to induce autoimmunity (8, 9). The authors concluded, however, that pre-existing MG is not an absolute contraindication for ICI treatment (10).

For paraneoplastic neurological diseases, cancer can be diagnosed within 2 years of the onset of the neurological disorder (11). Paraneoplastic syndromes are present in more than 8% of cancer patients. The incidence has increased over time, which is likely associated with better diagnostic approaches and longer survival of these patients (12). Many paraneoplastic neurological syndromes are immune-mediated and associated with circulating autoantibodies to a number of known antigens. For paraneoplastic dermatomyositis, they include TIF1-γ (present in patient 1) and nuclear matrix protein-2 (NXP-2, also known as anti-MJ or p140). For paraneoplastic neuropathies, the following antibodies have been identified: Hu (also known as anti-neuronal nuclear antibody type 1, ANNA-1), CV2/CRMP-5, ganglionic acetylcholine receptor, and MAG (8–10). For other neuromuscular diseases, common autoantibodies associated with paraneoplastic syndromes include amphiphysin antibody, glial nuclear antibody, voltage-gated potassium channel antibody, P/Q-type calcium channel antibody, and Purkinje cell cytoplasmic antibody. For many cases, CSF results are positive when serum results are negative and are more readily interpretable because CSF generally lacks the interfering nonorgan-specific antibodies that are common in the serum of patients with cancer. The cancers may be new or recurrent, are usually limited in metastatic volume, and are often occult by standard imaging procedures. Detection of the informative marker autoantibodies allows early diagnosis and treatment of the cancer, which may lessen neurological morbidity and improve patient survival. However, up to 30% of the cases may not have any of the previously identified paraneoplastic autoantibodies, the so-called seronegative paraneoplastic cases, making the diagnosis of paraneoplastic syndrome challenging. For involvement of the peripheral nerves or muscles, the clinical presentations may be similar to GBS (an acute inflammatory polyneuropathy), CIDP, autoimmune autonomic ganglionopathy, and inflammatory myopathy (11–14). A population-based cohort study in Denmark suggested that GBS patients had a three fold increased risk of cancer diagnosis in the first year of follow-up with an absolute cancer risk of 2.7% (15). Some cases with paraneoplastic neuromuscular disorders respond to immunosuppressants or immune modulators including IVIG (12). In these cases, when treatment with ICI is considered, there is concern that the immunosuppressants/immunomodulators will reduce the effectiveness of the ICI, making the treatment of cancer patients with pre-existing paraneoplastic syndromes even more complicated.

In the patients reported here, we had concluded, given the temporal association with the onset of underlying neoplasm, that each had paraneoplastic neuromuscular diseases because the cancers were diagnosed within 2 years of the onset of the neurological symptoms and the improvements of neuromuscular diseases correlated with the reduction of the tumors (11). Case 1 had paraneoplastic dermatomyositis and patient 2 had “seronegative” paraneoplastic demyelinating neuropathy (11, 12). The first case had clinical characteristics of dermatomyositis with positive TIF1-γ antibody, which has high specificity for cancer-associated dermatomyositis in adults. TIF1-γ is involved in the TGF-β signaling pathway, which is important for tumorigenesis and metastasis (16). The patient experienced the rash before the rapidly growing neck mass appeared. The dual treatment with PD-1 inhibitor and IVIG for 11 months resulted in the clearing of the clinical findings of dermatomyositis and normalization of the elevated serum CK, along with resolution of all the previously metabolic avid tumor lesions by PET scan, all of which support the diagnosis of paraneoplastic dermatomyositis.

The second case had been in remission from melanoma since surgical excision 27 years earlier and was found to have recurrent melanoma 1 year after the onset of demyelinating neuropathy. After 6 months of IVIG treatment alone, she had worsening neurological examination and EMG findings of polyneuropathy. Both her objective neurological exam and EMG studies improved along with the reduction in tumor size that followed combined ICI treatment and low-dose IVIG infusion for 12 months. The IVIG was discontinued at 52 months after the onset of initial neurological symptoms at which time the patient’s neurological exam had returned to normal and PET showed no evidence of cancer. Even after the patient had been off IVIG for 12 months, her neurological exam remained normal and the surveillance PET scan did not show any evidence of cancer.

Both patients received dual ICI and IVIG treatment without exacerbation of myopathy or neuropathy. Moreover, in each, the neurological symptoms/examination improved in conjunction with tumor reduction. The first patient had significant improvements of the rash and proximal extremity weakness at 15 months of symptom onset while the metastatic lung cancer lesions decreased in size. The second patient had continued progression of the neuropathy at 14 months after symptom onset, by neurological exam and EMG findings, despite standard IVIG treatment (initial dose 2 g/kg every 4 weeks followed by maintenance dose of 1 g/kg every 4 weeks). In contrast to the response to IVIG alone, addition of the ICI to treat the tumor resulted in improvement of the neuropathy by both neurological examination and electrodiagnostic parameters.

With the increasing use of ICI in cancer treatments, ICI-associated neuromuscular complications have been more frequently encountered in clinical practice (1, 2). Thus, the early detection of ICI-associated neuromuscular complications has been the recent focus of both oncologists and neurologists. However, the early diagnosis of ICI-associated neuromuscular complications has been challenging. The wide spectrum of ICI-associated neuromuscular complications overlaps with paraneoplastic neuromuscular diseases, including myositis, myasthenia syndromes, and CIDP-like neuropathy. Careful attention to the time course of the illness and serial comprehensive physical examinations are very important to help differentiate paraneoplastic neuromuscular disorders from ICI-associated neuromuscular complications. As we know, the onset of neuromuscular symptoms frequently precedes the diagnosis of cancers in paraneoplastic neuromuscular disorders. On the other hand, the onset of neuromuscular symptoms follows the ICI treatment in cancer patients with ICI-associated neuromuscular complications. The differentiation between these two different categories of neuromuscular diseases will be very important for clinicians to decide if the patients should continue ICI or immunosuppressants or not.

The two patients presented here had excellent tumor responses to ICI along with improvement of the neuromuscular diseases. Our results support the view that ICI is not contraindicated in cancer patients with pre-existing immune-mediated neuromuscular diseases. In both cases, the neuromuscular disorders presented as paraneoplastic manifestations of the underlying neoplasm. Combined treatment with ICI and immunomodulator IVIG helped control the neuromuscular disease in each case, without compromising the anti-tumor effect of ICI. However, we recommend close monitoring of both the neuromuscular disease and the cancer during such treatment.

## Data availability statement

The original contributions presented in the study are included in the article/supplementary material. Further inquiries can be directed to the corresponding author.

## Ethics statement

The studies involving human participants were reviewed and approved by the IRB committee of the University of California, Davis (IRB ID 1784252-1) with the waiver of patients’ consent. Written informed consent was obtained from the participant/patient(s) for the publication of this case report.

## Author contributions

GX: Initiate the draft of the manuscript, summarize the two cases, contribute to the figures, **Tables 1**, **2** and part of discussion. RY: Contribute to the figures, case history and managements of patient 1. HC: Contribute to the case history and management of patient 2, part of **Table 1**. EM: Contribute to the case history and management of patient 1, part of the **Figure 1**. RM: Contribute to part of the case history and management of patient 2. DR: Contribute to part of case summaries and discussion. TL, MD: Contribute to the figures, introduction and part of discussion, **Figures 1**, **2**. All authors contributed to the article and approved the submitted version.

## References

[1] Kao JC Liao B Markovic SN Klein CJ Naddaf E Staff NP. Neurological complications associated with anti-programmed death 1 (PD-1) antibodies. JAMA Neurol (2017) 74(10):1216–22. doi: 10.1001/jamaneurol.2017.1912 PMC5710300 28873125

[2] Chompoopong P Zekeridou A Shelly S Ruff M Dyck PJ Klein CJ. Comparison of immune checkpoint inhibitor-related neuropathies among patients with neuroendocrine and non-neuroendocrine tumours. J Neurol Neurosurg Psychiatry (2021) 93(1):112–4. doi: 10.1136/jnnp-2021-326369 34039631

[3] Collins L Quinn A Stasko T. Skin cancer and immunosuppression. Dermatol Clin (2019) 37(1):83–94. doi: 10.1016/j.det.2018.07.009 30466691

[4] Dias Lopes NM Mendonça Lens HH Armani A Marinello PC Cecchini AL. Thyroid cancer and thyroid autoimmune disease: a review of molecular aspects and clinical outcomes. Pathol Res Pract (2020) 216(9):153098. doi: 10.1016/j.prp.2020.153098 32825964

[5] Zhou Z Liu H Yang Y Zhou J Zhao L Chen H. The five major autoimmune diseases increase the risk of cancer: epidemiological data from a large-scale cohort study in China. Cancer Commun (Lond). (2022) 42(5):435–46. doi: 10.1002/cac2.12283 PMC9118055 35357093

[6] Dunn GP Bruce AT Ikeda H Old LJ Schreiber RD.. Cancer immunoediting: from immunosurveillance to tumor escape. Nat Immunol (2002) 3:991–8. doi: 10.1038/ni1102-991 12407406

[7] Liu X Hogg GD DeNardo DG. Rethinking immune checkpoint blockade: 'Beyond the T cell'. J Immunother Cancer. (2021) 9(1):e001460. doi: 10.1136/jitc-2020-001460 33468555 PMC7817791

[8] Ramos-Casals M Brahmer JR Callahan MK Flores-Chávez A Keegan N Khamashta MA. Immune-related adverse events of checkpoint inhibitors. Nat Rev Dis Primers. (2020) 6(1):38. doi: 10.1038/s41572-020-0160-6 32382051 PMC9728094

[9] Khan S Gerber DE. Autoimmunity, checkpoint inhibitor therapy and immune-related adverse events: a review. Semin Cancer Biol (2020) 64:93–101. doi: 10.1016/j.semcancer.2019.06.012 31330185 PMC6980444

[10] Narayanaswami P Sanders DB Wolfe G Benatar M Cea G Evoli A. International consensus guidance for management of myasthenia gravis: 2020 update. Neurology (2021) 96(3):114–22. doi: 10.1212/WNL.0000000000011124 PMC7884987 33144515

[11] Graus F Delattre JY Antoine JC Dalmau J Giometto B Grisold W. Recommended diagnostic criteria for paraneoplastic neurological syndromes. J Neurol Neurosurg Psychiatry (2004) 75(8):1135–40. doi: 10.1136/jnnp.2003.034447 PMC1739186 15258215

[12] Pelosof LC Gerber DE. Paraneoplastic syndromes: an approach to diagnosis and treatment. Mayo Clin Proc (2010) 85(9):838–54. doi: 10.4065/mcp.2010.0099 PMC2931619 20810794

[13] Koike H Tanaka F Sobue G. Paraneoplastic neuropathy: wide-ranging clinicopathological manifestations. Curr Opin Neurol (2011) 24(5):504–10. doi: 10.1097/WCO.0b013e32834a87b7 21799410

[14] Baijens LW Manni JJ. Paraneoplastic syndromes in patients with primary malignancies of the head and neck: four cases and a review of the literature. Eur Arch Otorhinolaryngol (2006) 263:32–6. doi: 10.1007/s00405-005-0942-1 15986184

[15] Girma B Farkas DK Laugesen K Skajaa N Henderson VW Boffetta P. Cancer diagnosis and prognosis after Guillain-Barré syndrome: a population-based cohort study. Clin Epidemiol. (2022) 14:871–8. doi: 10.2147/CLEP.S369908 PMC9309322 35898330

[16] De Vooght J Vulsteke JB De Haes P Bossuyt X Lories R De Langhe E. Anti-TIF1-γ autoantibodies: warning lights of a tumour autoantigen. Rheumatol (Oxford). (2020) 59(3):469–77. doi: 10.1093/rheumatology/kez572 31883334

